# COVID-19 rapid antigen testing strategies require careful evaluation

**DOI:** 10.1016/j.ebiom.2021.103491

**Published:** 2021-07-17

**Authors:** Jacqueline Dinnes

**Affiliations:** Test Evaluation Research Group, Institute of Applied Health Research, University of Birmingham, UK

Throughout the Covid-19 pandemic, testing for SARS-CoV-2 to allow early identification and isolation of those likely to be infectious has been a cornerstone of public health strategies to interrupt transmission of infection. An early reliance on reverse transcription polymerase chain reaction (RT-PCR) to confirm infection in those showing ‘typical’ signs and symptoms associated with Covid-19 was quickly supplemented with the use of rapid antigen detection tests (RDTs), particularly as the use case scenarios for testing changed from a focus on containment to a situation where testing has a much wider role as part of efforts to ease societal restrictions [1]. The need for specialist laboratory facilities and worldwide demand for reagents limited countries’ ability to quickly scale up RT-PCR testing [2]. RDTs conversely, are less expensive, provide results significantly more quickly, and do not require the same technical expertise or specialist facilities making them attractive for wide scale deployment [1].

The advantages of RDTs come at a cost of lower and more variable sensitivity. Rt-PCR (able to detect even the smallest amount of viral RNA) is highly sensitive for detection of SARS-CoV-2, but has been criticised for its continued detection of the virus long after an individual is likely to remain infectious [3]. RDTs use antibodies to capture SARS-CoV-2 proteins and accuracy is strongly affected by viral load. Indeed, test manufacturers have typically validated RDTs for use in symptomatic populations during the first five to seven days after symptom onset when viral load is expected to be highest and, by corollary, individuals are more likely to be infectious. A Cochrane review has shown RDT sensitivity of 78% on average during the first week after onset of symptoms (based on 2,320 RT-PCR positive cases), however observed sensitivities varied considerably, in part because of differences between assays, but also likely to reflect differences in sample types and storage, the adequacy of sampling and test interpretation [4]. The same review highlighted the need for more information about how well RDTs perform in those who do not show typical signs and symptoms of Covid-19, and the lack of evidence for mass testing of asymptomatic individuals with no epidemiological indication for testing, for example to allow return to work or school, facilitate travel, or allow resumption of mass gatherings [4].

In this article of *EBioMedicine* [5], Isabell Wagenhäuser and colleagues provide a significant contribution to the evidence base for targeted community-based screening with RDTs. The ‘community’ consisted of over 5000 participants at a tertiary care hospital facility including all admitted patients or those accompanying patients, and hospital employees with respiratory symptoms or close contact with confirmed cases. The observed sensitivity of the RDT testing strategy was low – 42% overall and rising only to 49% in those with a ‘typical’ Covid-19 symptom profile. Almost all false positive results (15/16) occurred in those with no or with atypical Covid-19 symptoms, so that nearly three quarters of positive RDT results in this group were falsely positive (positive predictive value of 29%). As expected, the tests performed better in those with higher viral load and the authors point to the ability of the tests to detect all samples from individuals who may be considered ‘super spreaders’ of infection (detecting all 8 samples with SARS-CoV-2 RNA copies/mL of 10^8 or above), a claim that is supported by empirical studies of transmission risks [6]. However, transmission of infection does not stop at any specific viral load cut-off but operates on a continuum, with transmission also occurring in those with much lower viral loads [6]. Considering samples with viral loads above the commonly advocated cut-off for ‘infectiousness’ of 106 RNA copies/mL [7], 6 of 32 likely infectious cases in this study were missed.

Wagenhäuser and colleagues[5] reported RDT sensitivity at the lower end of that observed in other recent large-scale studies of RDT testing strategies which also included large proportions of asymptomatic people [8,9], so what are the possible explanations? Between-assay variations in sensitivity and the use of possibly less sensitive oropharyngeal samples may well have contributed [10], however the low prevalence of SARS-CoV-2 and differences in the case-mix of participants, particularly in terms of the distribution of viral loads are likely to be a driving factor. While this study applied a blanket testing policy regardless of indication for SARS-CoV-2 testing, studies conducted at freely available Covid-19 testing sites will include those sufficiently motivated to attend and a greater proportion of people with epidemiological indications for testing [8,9]. The study period also overlapped a strict lockdown with declining prevalence of infection, and consequently lower proportions of people with the highest viral loads.

The ability of RDTs to quickly pick up the majority of individuals with high levels of virus is undeniable but these tests do still miss people who are likely to be infectious and also risk falsely classifying uninfected individuals as positive. RDTs alone cannot be relied on to prevent outbreaks but, used in combination with Covid-19 symptom profiles and as triage to RT-PCR, can be a useful component of strategies to allow routine hospital procedures to continue. This study clearly emphasises the need for careful evaluation of RDT testing strategies for any given use case scenario prior to widespread roll-out.

## Contributors

JD was solely responsible for the writing of the commentary.

## Declaration of Competing Interest

The author declares no conflict of interest.
